# Identification of biomarkers regulated by rexinoids (LGD1069, LG100268 and Ro25-7386) in human breast cells using Affymetrix microarray

**DOI:** 10.3892/mmr.2015.3480

**Published:** 2015-03-12

**Authors:** HYE-SOOK SEO, JONG-KYU WOO, YONG CHEOL SHIN, SEONG-GYU KO

**Affiliations:** 1 Laboratory of Clinical Biology and Pharmacogenomics and Center for Clinical Research and Genomics, College of Korean Medicine, Kyung Hee University, Dongdaemun-gu, Seoul 130-701, Republic of Korea; 2 Laboratory of Preventive Pharmacy, College of Pharmacy, Gachon University of Medicine and Science, Yeonsu-gu, Incheon 406-840, Republic of Korea

**Keywords:** breast cancer, rexinoid, biomarker, LGD1069, LG100268, Ro25-7386

## Abstract

Retinoids possess anti-proliferative properties, which suggests that they possess chemopreventive and therapeutic potential against cancer. In the current study, genes modulated by rexinoids (retinoid X receptor (RXR)-pan agonists, LGD1069 and LG100268; and the RXRα agonist, Ro25-7386) were identified using an Affymetrix microarray in normal and malignant breast cells. It was observed that LGD1069, LG100268 and Ro25-7386 suppressed the growth of breast cells. Secondly, several rexinoid-regulated genes were identified, which are involved in cell death, cell growth/maintenance, signal transduction and response to stimulus. These genes may be associated with the growth-suppressive activity of rexinoids. Therefore, the identified genes may serve as biomarkers and novel molecular targets for the prevention and treatment of breast cancer.

## Introduction

Breast cancer is the most commonly diagnosed type of cancer in females and is the leading cause of cancer-related mortality in females worldwide (1). In 2013, the American Cancer Society estimated that 232,340 females would be newly diagnosed with breast cancer and 39,620 females would succumb to the disease (2). The key objectives of scientists and clinicians in managing this breast cancer are to prevent the incidence, detect it early and treat it with effective therapeutic strategies resulting in long overall survival with minimal side effects. Therefore, the aim of the current study was to identify the genes associated with cell growth inhibition that are induced by Retinoid X receptor (RXR)-selective retinoids (rexinoids), with an aim to improve prevention and treatment of breast cancer.

Retinoids regulate a variety of biological functions, including embryogenesis, growth, differentiation, vision and reproduction (3–5). Retinoids additionally possess antiproliferative properties, which suggests a chemopreventive and therapeutic role against cancer (6). In addition, retinoids have been reported to inhibit normal- or tumor-cell growth through the regulation of differentiation and/or apoptosis (7–10).

Retinoids exert their effects in target cells via interaction with retinoic acid receptors (RARs) and RXRs. Each of these includes three subtypes, termed α, β and γ, which are encoded by distinct genes. The RARα, RARβ and RARγ genes have been localized to chromosomes 17q21, 3p24 and 12q13, respectively. The RXRα, RXRβ and RXRγ genes have been mapped to chromosomes 9q34.3, 6p21.3 and 1q22-23, respectively (11). The RARs bind all-*trans*-retinoic acid (ATRA) and 9-*cis*-retinoic acid (RA) while RXRs bind 9-*cis*-RA alone. RXRs are known to heterodimerize with several steroid hormone receptors, including RAR, thyroid hormone receptor, vitamin D receptor, peroxisome proliferator-activated receptor, liver X receptor, pregnane X receptor and farnesoid X receptor suggesting its involvement in several signaling pathways (12). RXRs are also able to homodimerize in transfected cells (13).

In addition to naturally occurring retinoids, including ATRA, 9-*cis*-RA and 13-*cis*-RA, various synthetic retinoids with varied selectivity have been developed and are currently available to treat psoriasis, acne, photoaging, actinic keratosis and certain types of cancer, including acute promelocytic leukemia, cutaneous T-cell lymphoma and squamous or basal cell carcinoma (14). However, the use of RAR-selective retinoids is limited by their toxicity, which can result in chelitis, hypertriglyceridemia and hepatosplenomegaly (15).

Rexinoids are important in controlling apoptosis and can function in a ligand-dependent or ligand-independent manner (16,17). Notably, rexinoids have been reported to suppress estrogen receptor (ER)-positive and ER-negative mammary tumor development with reduced toxicity compared with RAR-selective retinoids (18–20). Rexinoids are additionally active in animals with tamoxifen-resistant breast cancer (17,21) and in ATRA-resistant breast cancer cells (22). Thus, rexinoids appear to be promising chemopreventive and therapeutic agents with improved efficiency as compared with RAR-selective ligands. Among the rexinoids, LGD1069 (Bexarotene) was confirmed as a safe and well-tolerated agent in clinical trials of cutaneous T-cell lymphoma, breast cancer and lung cancer (22,23).

Thus, we focussed on rexinoids and their cognate receptor, RXR, in breast cells, and aimed to investigate their regulatory activity on the transcription of genes involved in growth suppression. In particular, the present study investigated the RXRα isoform, which has been suggested as a potential therapeutic target in breast cancer cells, due to the observation that overexpression of RXRα sensitized breast cancer cells lines to the antiproliferative effects of RXR-selective ligands (24). In addition, infection with adenoviral RXRα induced nucleoplasmic overexpression of RXRα and resulted in apoptosis with treatment with an RXR ligand in retinoid-resistant MDA-MB-231 cells (25). Thus, in the current study, the growth-suppressive activity of RXR pan agonists (LGD1069 and LG100268) and an RXRα specific ligand (Ro25-7386) were investigated in normal human mammary epithelial cells (HMECs) and four breast cancer cell lines (MCF-7, T47D, MDA-MB-231 and MDA-MB-435) using an MTS assay. Subsequently, the genes regulated by rexinoids that may be involved in their antiproliferative activity were investigated with an Affymetrix microarray.

## Materials and methods

### Ligands and antibodies

LGD1069 and LG100268 were provided by Ligand Pharmaceuticals, Inc. (La Jolla, CA, USA). Ro25-7386 was obtained from Roche Bioscience (Palo Alto, CA, USA). These compounds were diluted in dimethyl sulfoxide purchased from Sigma-Aldrich (St. Louis, MO, USA) to a final concentration of 0.1%. Monoclonal or polyclonal antibodies (mouse or rabbit) against RXRα (cat. no. sc-553) B-cell lymphoma 2-associated X protein (Bax; cat. no. sc-7480), E-cadherin (cat. no. sc-7870), integrin α6 (cat. no. sc-13542), cell division control protein 42 (CDC42; cat. no. sc-8401) and actin (cat. no. sc-8432) were purchased from Santa Cruz Biotechnology, Inc. (Santa Cruz, CA, USA).

### Cells and culture materials

Human normal mammary epithelial cells (HMECs) were obtained from Lonza Group (San Diego, CA, USA). Cells between passages 10 and 11 were used for experiments and the cells were grown and maintained in mammary epithelial basal medium supplemented with 13 mg/ml bovine pituitary extract, 0.5% serum, 5 *μ*g/ml insulin, 10 ng/ml human recombinant epidermal growth factor, 0.5 mg/ml hydrocortisone, 50 *μ*g/ml gentamicin and 50 *μ*g/ml amphotericin-β (all Clonetics, Lonza Group, San Diego, CA, USA). Cells were maintained in a humidified environment at 37°C with 5% CO_2_ in air.

Four different human breast cancer cell lines (MCF-7, T47D, MDA-MB-231 and MBA-MB-435) purchased from the American Type Culture Collection (Manassas, VA, USA) were grown and maintained in appropriate growth media; minimal essential medium for MCF-7 and RPMI 1640 for T47D, MDA-MB-231 and MBA-MB-435 (Invitrogen Life Technologies, Carlsbad, CA, USA) supplemented with 10% heat-inactivated fetal bovine serum (FBS; Welgene, Daegu, Korea). L-glutamine, penicillin, streptomycin and gentamicin (Life Technologies Korea, LLC, Seoul, Korea) were used at the usual concentrations. For all experiments, breast cancer cells were harvested by trypsinization (0.25% trypsin and 0.02% EDTA; Life Technologies Korea, LLC), seeded and grown in the appropriate media containing 10% FBS in a humidified 95% air 5% CO_2_ atmosphere.

### Cell growth rate measurements

The CellTiter 96^®^ AQ_ueous_ Non-Radioactive Cell Proliferation Assay (Promega Corporation, Madison, WI, USA) was used for the measurement of cell growth rate in breast cancer cells according to the manufacturer’s instructions. The CellTiter 96^®^ AQ_ueous_ Assay is composed of solutions of a novel tetrazolium compound [3-(4,5-dimethylthiazol-2-yl)-5-(-carboxymethoxyphenyl)-2-(4-sulfophenyl)-2H-tetrazolium, inner salt; MTS] and an electron coupling reagent (phenazine methosulfate; PMS). Briefly, HMECs, MCF-7 and T47D (1,000 cells/well) were plated in 96-well plates. Following a 24 h resting period, LGD1069, LG100268 and Ro25-7386 were added into the growth media and cell culture continued for 8–12 days. Each measurement day (every 2 days), MTS (Promega Corporation) was added to the cells (20 *μ*l combined MTS/PMS solution per 100 *μ*l culture medium) and further incubation was conducted for 2 h. MTS is bioreduced by cells into a formazan product that is soluble in tissue culture medium. The absorbance of the formazan at 490 nm was measured directly using an ELISA plate reader (Gemini EM Microplate reader, Versa Max, Fluorescence readers; Molecular Devices, Sunnyvale, CA, USA). Each data point was performed in quadruplicate and the results were presented as the mean absorption (optical density).

### RNA target preparation/Affymetrix microarray analysis

Total RNA was extracted from different breast cells treated with rexinoids using the guanidinium isothiocynate method (TRIzol reagent; Invitrogen Life Technologies) followed by purification using an RNeasy column (Qiagen, Valencia, CA, USA). RNA quality was assessed using the 2100 Bioanalyzer Instrument (Agilent Technologies, Inc., Palo Alto, CA, USA). A total of 10 *μ*g total RNA was processed for use on the microarray using the Affymetrix GeneChip One-Cycle Target Labeling kit (Affymetrix, Inc., Santa Clara, CA, USA) according to the manufacturer’s instructions. The resultant biotinylated cRNA was fragmented and then hybridized to the Affymetrix U133 Plus 2.0 GeneChip. The arrays were washed, stained and scanned using the Affymetrix 450 Fluidics Station and GeneChip Scanner 3000 7G (Affymetrix, Inc.) according to the manufacturer’s recommendations. Expression values were generated using Microarray Suite software, version 5.0 (Affymetrix, Inc.).

### Statistical analysis of microarray data

Background subtraction and normalization using the robust multi-array average algorithm method was performed using GeneSpring GX 11.5 software (Agilent Technologies) for gene expression. Fold change values for genes were calculated as the ratio of the signal values of the experimental group compared with the control group. Alterations in gene expression >2-fold were considered to be statistically significant. Genes of interest were selected by referring to the PathArt program which shows intersection of genes in several signaling pathways.

### Reverse transcription-quantitative polymerase chain reaction (RT-qPCR) analysis

Cells were cultured to 80–90% confluence. Total RNA was prepared using the Qiagen RNeasy Mini kit (Qiagen). The RT reaction was performed using 1 *μ*g total RNA which was reverse-transcribed into cDNA using a random hexamer primer (GeneAmp RNA PCR Core kit; Applied Biosystems Life Technologies, Foster City, CA, USA), according to the manufacturer’s instructions. cDNA of the 7 selected genes and an internal reference gene (GAPDH) was produced from each sample and was quantified using a fluorescence-based real-time detection method (iCycler; Bio-Rad Laboratories, Inc., Hercules, CA, USA). RT-qPCR analysis was performed using the standard methods recommended by the RT-qPCR kit supplier (SYBR^®^ Green Dye-Based Gene Expression Detection; Applied Biosystems Life Technologies). Primer sequences used for detection of RXRα-regulated genes are shown in Table I (Cosmo Genetech, Seoul, Korea). For the endogenous control, human GAPDH labeled with VIC™ dye provided by Applied Biosystems Life Technologies was used. The amplification conditions were as follows: 30 sec at 95°C and 3 min at 95°C, and 30 sec at 95°C and 60 sec at 65°C for 40 cycles, followed by a final extension for 20 min at 72°C. The ratio between the values obtained provided the relative gene expression levels.

### Western blot analysis

Whole cell extracts were prepared using 1X sodium dodecyl sulfate (SDS) laemmlli lysis buffer (125 mM Tris-HCl, pH 6.8; 1% SDS; 2% β-mercaptoethanol). Total cell lysates with equal quantities of protein (30 *μ*g) were subjected to 10% SDS-PAGE and subsequently electrotransferred onto a nitrocellulose membrane (Bio-Rad Laboratories, Inc.). Membranes were blocked with 5% skimmed milk in PBST (phosphate-buffered saline containing 0.1% Tween 20) for 1 h at room temperature, then incubated overnight with primary antibodies in PBST containing 2.5% bovine serum albumin (1:1,000 dilution). Subsequent to washing with PBST, the blot was further incubated for 1 h at room temperature with peroxidase conjugated anti-rabbit or anti-mouse antibodies (Pierce Technology Corporation, Holmdel, NJ, USA) in PBST and then visualized using the enhanced chemiluminescence system (GE Healthcare Life Sciences, Chalfont, UK). Protein expression was normalized using β-actin expression.

### Statistical analysis

All experiments were performed in triplicate. Statistical analyses were performed using Microsoft Excel 2007 (Microsoft Corporation, Albuquerque, NM, USA). The data for the MTS assay and RT-qPCR are expressed as the mean ± standard deviation. Student’s t-test was used for single variable comparisons, and P<0.05 was considered to indicate a statistically significant difference.

## Results

### Anti-proliferative activity of rexinoids

**Figure 1:**
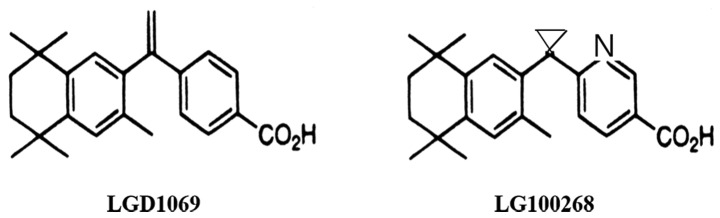
Molecular structure of LGD1069 and LG100268.

**Figure 2:**
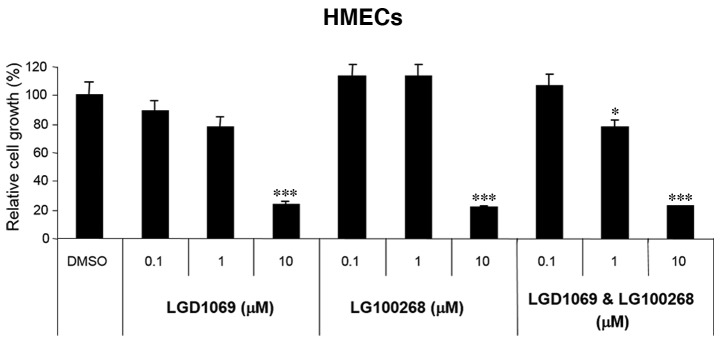
Effect of LGD1069 and LG100268 on the growth of HMECs. HMECs were treated with different doses of LGD1069 and/or LG100268 (0.1–10 *μ*M). The relative cell growth rate was measured by an MTS assay after 10 days. The growth rate of the vehicle-treated cells was set to 100% and the relative reduction in cell viability resulting from the treatment with rexinoids was expressed as a percentage of the control. Data are presented as the mean of three independent experiments (error bars denote the standard deviation; ^*^P<0.05, ^**^P<0.01 and ^***^P<0.001 vs. control). HMECs, human mammary epithelial cells; DMSO, dimethyl sulfoxide.

**Figure 3:**
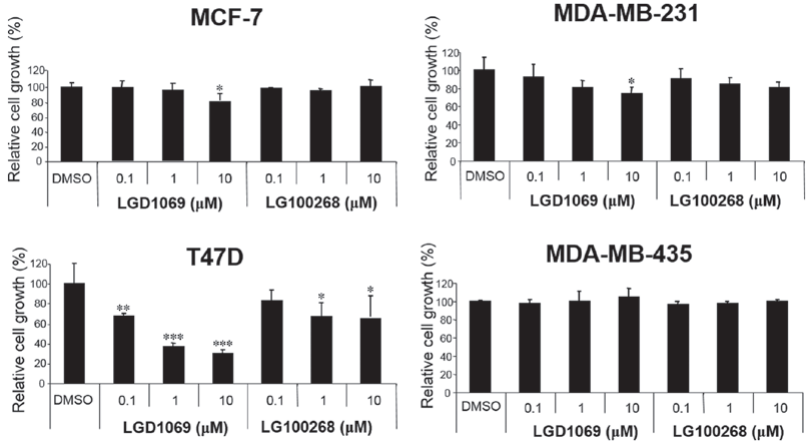
Effect of LGD1069 and LG100268 on the growth of breast cancer cells. MCF-7, T47D, MDA-MB-231 and MDA-MB-435 cells were treated with different doses of LGD1069 or LG100268 (0.1–10 *μ*M). The relative cell growth rate was measured by an MTS assay after 10 days. The growth rate of the vehicle-treated cells was set to 100%, and the relative reduction in cell viability resulting from the treatment with rexinoids was expressed as a percentage of the control. Data are presented as the mean of three independent experiments (error bars denote the standard deviation; ^*^P<0.05, ^**^P<0.01 and ^***^P<0.001 vs. control). DMSO, dimethyl sulfoxide.

In Fig. 1, the structures of LGD1069 and LG100268 are presented. The anti-proliferative effects of rexinoids in normal and malignant breast cells were investigated. It was observed that LGD1069 and LG100268 significantly suppressed cell growth in HMECs at 10 *μ*M; whereas co-treatment with LGD1069 and LG100268 reduced cell growth at 1 and 10 *μ*M suggesting that these two rexinoids possess the capacity to prevent mammary cell growth (Fig. 2). By contrast, LGD1069 weakly (10 *μ*M, P<0.05) inhibited cell growth in MCF-7 cells while the compound strongly and significantly suppressed cell growth in a dose-dependent manner in T47D cells (0.1 *μ*M, P<0.01; 1 and 10 *μ*M, P<0.001) (Fig. 3). Notably, LGD1069 induced mild inhibition (P<0.05) of cell growth in MDA-MB-231 cells at 10 *μ*M while rexinoids did not affect cell growth in MDA-MB-435 cells (Fig. 3). This result indicates that LGD1069 is able to inhibit the growth of ER-negative breast cancer with therapeutic potency.

**Figure 4:**
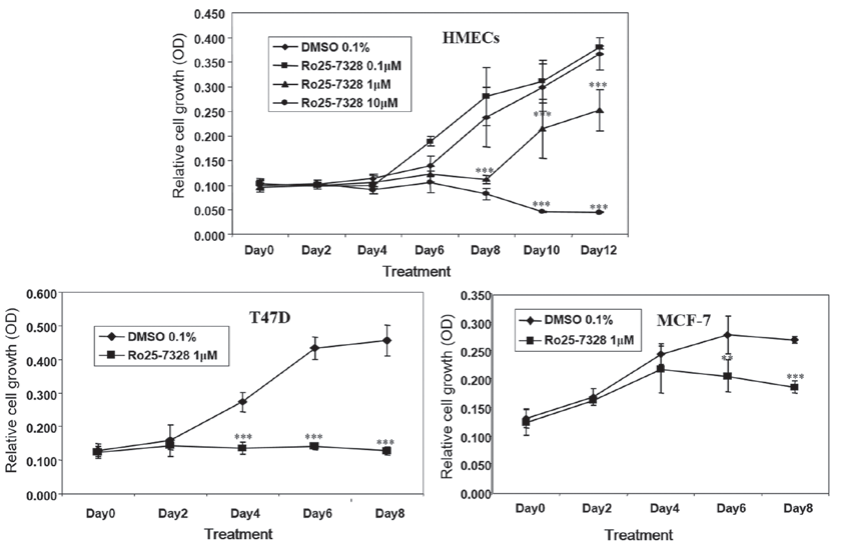
Effect of Ro25-7386 on the growth of breast cells. HMECs, MCF-7 and T47D cells were treated with Ro25-7386 for 0–12 days. The relative cell growth rate was measured by an MTS assay. Data are presented as the mean of three independent experiments (error bars denote the standard deviation; ^*^P<0.05, ^**^P<0.01 and ^***^P<0.001 vs. control). DMSO, dimethyl sulfoxide; OD, optical density.

In addition, Ro25-7386, the RXRα agonist significantly suppressed cell growth in a dose-dependent manner in HMECs. Ro25-7386 strongly reduced T47D cell growth at 1 *μ*M and induced suppression of cell growth in MCF-7 cells at day 8 at 1 *μ*M (Fig. 4). These results suggest that RXRα is important in the suppression of growth induced by rexinoids in breast cells.

### Expression of RXRα in breast cells

**Figure 5:**
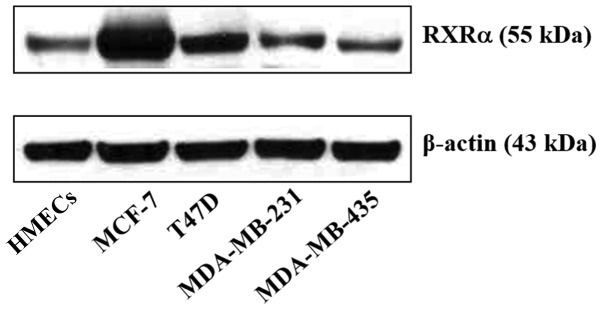
Expression levels of RXRα in normal and malignant breast cells. Whole cell lysates from normal (HMECs) and malignant breast cells (MCF-7, T47D, MDA-MB-231 and MDA-MB-435) were analyzed by western blotting with anti-RXRα. The data presented are representative of three independent experiments that gave similar results.

The RXRα level in normal and malignant breast cells was next determined. It was observed that all breast cell lines express RXRα but with different intensities. MCF-7 and T47D expressed higher levels of RXRα (Fig. 5). Notably, the ER-negative breast cancer cell lines, MDA-MB-231 and MDA-MB-435, also expressed RXRα.

### Identification of target genes regulated by rexinoids in normal and malignant breast cells by Affymetrix microarray

Finally, the genes regulated by rexinoids in normal (HMECs) and malignant (MCF-7, T47D and MDA-MB-231) breast cells were identified. Gene expression profiles were established using the Affymetrix microarray (human genome U133A 2.0). Among them, several genes involved in cell death, cell growth/maintenance, signal transduction and response to stimulus were identified.

In HMECs, 638 genes upregulated and 347 genes downregulated by Ro25-7386 with alterations in fold induction >2-fold were identified. A total of 22 genes were strongly upregulated (>10-fold) and 5 genes were strongly downregulated (>4-fold) in expression levels by Ro25-7386 (Table IIA and B). Among them, several genes were notable, including integrin β4, E-cadherin (CDH1), C-terminal binding protein 1 (CtBP1), integrin α6, paxillin (PAX), BAX, forkhead box O3A (FOXO3A) and signal transducer and activator of transcription 3 (STAT3) (upregulated genes), and collagen type VI α3 and cell division cycle 42 (CDC42) (downregulated genes).

In MCF-7 cells, 83 genes were upregulated and 98 genes were downregulated by Ro25-7328 with alterations in fold induction >2-fold were identified (Table III). Among them, several genes were recognized including transforming growth factor β2, immunoglobulin heavy constant γ1, protein kinase Cδ binding protein, interleukin 6 receptor and neurophilin 2 (upregulated genes), and cathepsin S, zinc finger protein 36, integrin β4, transforming growth factor β1, PAX and CtBP1 (downregulated genes).

In T47D cells, 16 upregulated genes and 3 downregulated genes modulated by LGD1069 were observed (Table IV), whereas 3 upregulated genes and 5 downregulated genes were identified to be modulated by LG100268 (Table V) with alterations in fold induction >2-fold. According to the data, several notable genes induced by LGD1069 and LG100268 in T47D cells were identified, including cytochrome P450, dehydrogenase/reductase member 3, metallothionein, neuro-oncological ventral antigen 1 and regulator of G-protein signaling 1 (for LGD1069), and chemokine, glutamate receptor, colon carcinoma-related protein and insulin-like growth factor binding protein 7 (for LG100268). In addition, 3 upregulated genes and 5 downregulated genes by Ro25-7386 were identified with alterations in fold induction >2-fold in T47D cells. Among them, chemokine (upregulated genes), and glutamate receptor, ionotropic kainite 2, colon carcinoma-related protein, insulin-like growth factor binding protein 7 and growth differentiation factor 8 were identified (Table VI).

In MDA-MB-231 cells, a total of 335 upregulated genes and 320 downregulated genes modulated by LGD1069 were observed (Table VII); whereas 118 upregulated genes and 432 downregulated genes were modulated by LGD100268 (Table VIII) with alterations in fold induction >2-fold. According to the data, several notable genes were identified, including several types of hypothetical protein, zinc finger homeobox 1b, recombination activating gene 2 and tumor protein D52 (for LGD1069), and zinc finger protein 21, Mdm2, and gonadotropin-releasing hormone 1 (for LG100268).

### Confirmation of the alterations of modulation of RXRα target genes of HMECs by RT-qPCR and western blot analysis

**Figure 6:**
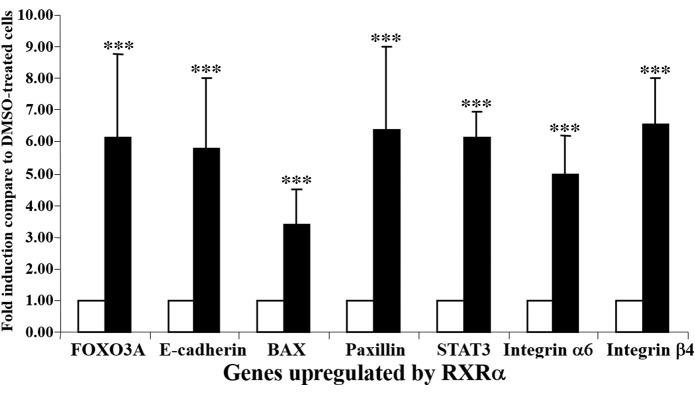
mRNA expression levels of RXRα-regulated genes in HMECs (measurement by RT-qPCR). HMECs were treated with Ro25-7386 (1 *μ*M) for 12 h. Subsequent to treatment, total RNA samples were extracted and RNA samples were subject to RT-qPCR analysis. Data are presented as the mean of three independent experiments (error bars denote the standard deviation; ^*^P<0.05, ^**^P<0.01 and ^***^P<0.001 vs. control). RT-qPCR, reverse transcription-quantitative polymerase chain reaction; DMSO, dimethyl sulfoxide.

**Figure 7:**
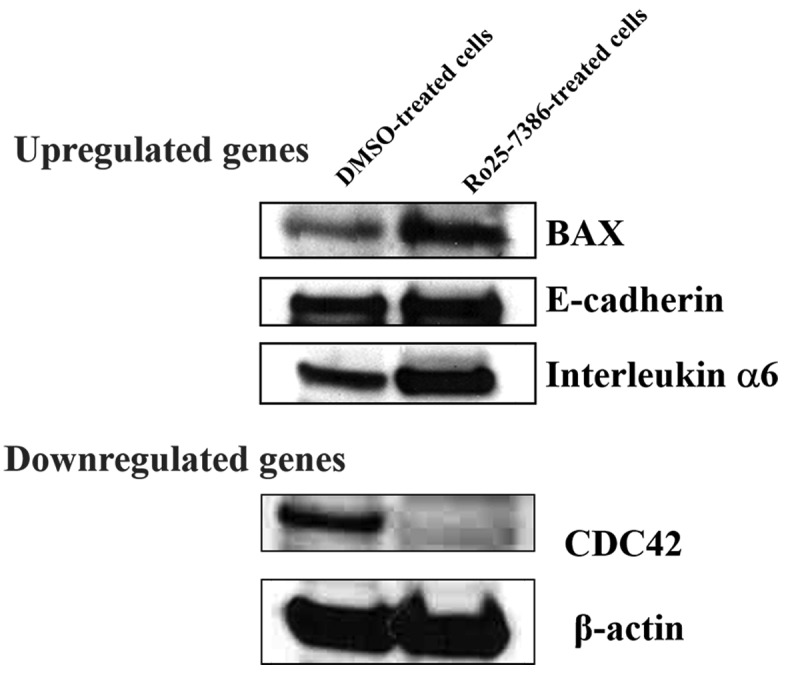
Protein levels of RXRα-regulated genes in HMECs measured by western blot analysis. HMECs were treated with Ro25-7386 (1 *μ*M) for 24 h. Subsequent to treatment, whole cell lysates were analyzed with anti-BAX, anti-E-cadherin, anti-interleukin α6 and anti-CDC42 antibodies. The data presented are representative of three independent experiments that gave similar results.

The induction of a total of 7 genes by rexinoid (mRNA levels) was confirmed by RT-qPCR assays. These 7 genes are as follows: Integrin β4, integrin α6, CDH1, PAX, BAX, FOXO3A and STAT3; and upregulation of these genes by Ro25-7386 was confirmed as demonstrated in Fig. 6. The alterations in fold induction of protein levels of certain genes were confirmed by western blot analysis; thus upregulation of BAX, CDH1, interleukin α6 and the downregulation of CDC42 is shown in Fig. 7.

Thorough investigation of the notable genes-CDH1, FOXO3A, BAX (HMEC-Ro25-7386), insulin-like growth factor binding protein 7 and growth differentiation factor 8 (T47D-Ro25-7386) and cathepsin S, TGFβ2, basigin, MCL-1 and BCL2L1 (MCF-7-Ro25-7386), may aid in the clarification of how RXRα agonists function to inhibit breast cell growth. Such notable genes are implicated in breast cancer management and are important for the treatment of breast cancer. The current study may aid in the elucidation of novel preventive/therapeutic targets for breast cancer, and may contribute to the development of novel molecules, which may be able to inhibit breast cancer development.

## Discussion

In order to investigate the molecular mechanism by which retinoids suppress breast cancer development, the current study focused upon RXR-specific ligands (rexinoids). These have been reported to suppress breast cancer development with minimal toxicity compared with RAR-specific ligands (21), and it was the RXRα isoform that was specifically focused upon in the present study that serves an important role in tumor suppression.

The human RXRα gene spans over 40 kilobases in size and consists of a minimum of 10 exons separated by introns ranging in size from 700 base pairs (intron 3) to >7.8 kb (intron 4) (26). It was observed that all of the cell lines examined expressed RXRα. Notably, ER-negative breast cancer cells, which do not respond to retinoid treatment, such as MDA-MB-231 and MDA-MB-435 also expressed RXRα. This suggests that RXRα is non-functional, losing DNA binding activity or failing to recruit essential co-activators required for the activation of the gene in ER-negative cells. Different and inappropriate sub-localization of the receptor may also explain the unresponsiveness of the cells to retinoid treatment.

LGD1069, LG100268 and Ro25-7386 were observed to suppress the growth of breast cells, including the normal HMECs and ER-positive breast cancer cells (MCF-7 and T47D). LGD1069 was observed to induce a mild inhibition of MDA-MB-231 cell growth at a dose of 10 *μ*M. LG100268 did not affect the cell growth as compared with LGD1069 in all four breast cancer cell lines suggesting its weaker activity. This result indicates that LGD1069 may possess the ability to inhibit the growth of ER-negative breast cancer.

The genes of interest were selected by referring to the PathArt program, which demonstrated the association between genes of several signaling pathways (data not shown). The alterations in gene expression were then analyzed using the Affymetrix microarray (human genome U133A 2.0) to determine which genes are associated with the inhibition of cell growth induced by the rexinoids. Among them, several genes were identified that are involved in cell death, cell growth/maintenance, signal transduction and response to stimulus, including E-cadherin, CtBP1, integrin β4, integrin α6, PAX, BAX, FOXO3A, STAT3, collagen type VI α3 and CDC42. It was additionally confirmed that Ro25-7386 upregulates the mRNA expression levels of FOXO3A, E-cadherin, BAX, PAX, STAT3, integrin α6 and integrin β4. In addition, Ro25-7386 was observed to increase the levels of BAX, E-cadherin and integrin α6 but reduce the level of CDC42. These results suggest that RXRa may have a role in the prevention and treatment of breast cancer development.

Further investigation regarding the functions of selected genes may aid in the elucidation of novel preventive/therapeutic targets for breast cancer, and may additionally contribute to the development of novel molecules, which may inhibit breast cancer progression.

## Figures and Tables

**Table I:** Forward and reverse primers for amplification of targeted genes with reverse transcription-quantitative polymerase chain reaction.

Target gene	Forward primer	Reverse primer
BAX	5′-TGGAGCTGCAGAGGATGATTG-3′	5′-GAAGTTGCCGTCAGAAAACATG-3′
E-cadherin	5′-CACTGCCAACTGGCTGGAG-3′	5′-GGGTTAGCTCAGCAGTAAAG-3′
FOXO3A	5′-TCAATCAGAACTTGCTCCACCA-3′	5′-GGACTCACTCAAGCCCATGTTG-3′
Integrin α6	5- TTTCCCGTTTCTTTCTTGAGTTGT-3′	5′-TGGAAAAGGTAACTTGTGAGCCA-3′
Integrin β4	5-TTCCAAATCACAGAGGAGAC-3	5-CTTGAGGTTGTCCAGATCAT-3′
PXN	5′-TGGCTTCGCTGTCGGATTTC-3′	5′GTCAAGGGCTGTCACCACTTTATC-3′
PTEN	5′-AGAGCGTGCAGATAATGACAAG-3′	5′-GGATCAGAGTCAGTGGTGTCAG-3′
STAT	5′-CTGCTGCGGTTCAGTGAGAG-3′	5′-CCAAGTGAAAGTGACCCCTCC-3′
Collagen type VI α3	5′-CTGGGCAGACATACCATGTG-3′	5′-GCAAGTTCCTTCGTCTTTCG-3′

**Table II:** Genes up- and downregulated by Ro25-7386 in human mammary epithelial cells.

A, Genes upregulated by Ro25-7386		
Probe set	Gene	Fold change
213872_at	gb:BE465032/DB_XREF=gi:9510807/DB_XREF=hv76g09.×1/CLONE= IMAGE:3179392/FEA=EST/CNT=34/TID=Hs.173685.1/TIER=Stack/STK=15/UG=Hs. 173685/LL= 81688/UG_GENE=FLJ12619/UG_TITLE=hypothetical protein FLJ12619	27.55
204989_s_at	Integrin, β4	26.60
210317_s_at	Tyrosine 3-monooxygenase/tryptophan 5-monooxygenase activation protein, epsilon polypeptide	22.14
200935_at	Calreticulin	21.26
201130_s_at	Cadherin 1, type 1, E-cadherin (epithelial)	20.66
201123_s_at	Eukaryotic translation initiation factor 5A	19.04
200751_s_at	Heterogeneous nuclear ribonucleoprotein C (C1/C2)	18.97
214007_s_at	PTK9 protein tyrosine kinase 9	17.37
203392_s_at	C-terminal binding protein 1	16.35
204427_s_at	Coated vesicle membrane protein	16.00
216971_s_at	Plectin 1, intermediate filament binding protein 500 kDa	15.17
217211_at	Consensus includes gb:D50604/DEF=Human β-cytoplasmic actin (ACTBP9) pseudogene/FEA=CDS/DB_XREF=gi:2094759/UG=Hs.248007 Human β-cytoplasmic actin (ACTBP9) pseudogene	14.35
215780_s_at	SET translocation (myeloid leukemia-associated)	12.51
201971_s_at	ATPase, H^+^ transporting, lysosomal 70 kDa, V1 subunit A	11.75
204426_at	Coated vesicle membrane protein	11.74
220494_s_at	gb:NM_018678.1/DEF=Homo sapiens lipopolysaccharide specific response-68 protein (LSR68), mRNA./FEA=mRNA/GEN=LSR68/PROD=lipopolysaccharide specific response-68 protein/DB_XREF=gi:8923914/UG=Hs.103189 lipopolysaccharide specific response-68 protein/	11.18
215177_s_at	Integrin, α6	10.97
215434_x_at	AG1	10.35
214693_x_at	Hypothetical protein MGC8902///AG1///hypothetical protein DJ328E19.C1.1///hypothetical protein LOC200030///hypothetical protein LOC348482	10.34
211905_s_at	Integrin, β4	10.34
201048_x_at	RAB6A, member RAS oncogene family	10.03
214701_s_at	Fibronectin 1	10.01
210092_at	Mago-nashi homolog, proliferation-associated (*Drosophila*)	9.74
212107_s_at	DEAH (Asp-Glu-Ala-His) box polypeptide 9	9.68
202118_s_at	Copine III	9.48
217234_s_at	Villin 2 (ezrin)	9.09
208853_s_at	Calnexin	7.59
201742_x_at	Splicing factor, arginine/serine-rich 1 (splicing factor 2, alternate splicing factor)	7.44
208750_s_at	ADP-ribosylation factor 1	7.31
203803_at	Prenylcysteine oxidase 1	7.31
211162_x_at	Stearoyl-CoA desaturase (δ-9-desaturase)	7.30
202856_s_at	Solute carrier family 16 (monocarboxylic acid transporters), member 3	7.26
200796_s_at	Myeloid cell leukemia sequence 1 (BCL2-related)	7.25
213606_s_at	Rho GDP dissociation inhibitor (GDI) α	7.25
201373_at	Plectin 1, intermediate filament binding protein 500kDa	7.19
208057_s_at	GLI-Kruppel family member GLI2	7.04
217294_s_at	Enolase 1, (α)	6.99
213875_x_at	Chromosome 6 open reading frame 62	6.93
91816_f_at	Ring finger and KH domain containing 1	6.90
200806_s_at	Heat shock 60 kDa protein 1 (chaperonin)	6.69
214845_s_at	Calumenin	6.66
211823_s_at	Paxillin	5.75
206665_s_at	BCL2-like 1	5.40
208637_x_at	Actinin, α1	5.11
208677_s_at	Basigin (OK blood group)	4.66
221499_s_at	Syntaxin 16	4.16
209226_s_at	Transportin 1	3.90
201752_s_at	Adducin 3 (γ)	3.90
200766_at	Cathepsin D (lysosomal aspartyl protease)	3.90
203085_s_at	Transforming growth factor, β1 (Camurati-Engelmann disease)	3.75
211833_s_at	BCL2-associated X protein	3.65
208852_s_at	Calnexin	3.49
210655_s_at	Forkhead box O3A	3.33

B, Genes downregulated by Ro25-7386		
Probe set	Gene	Fold change

203991_s_at	Ubiquitously transcribed tetratricopeptide repeat, X chromosome	−5.32
220568_at	gb:NM_018582.1/DEF=Homo sapiens hypothetical protein PRO1483 (PRO1483), mRNA./FEA=mRNA/GEN=PRO1483/PROD=hypothetical protein PRO1483/DB_XREF=gi:8924047/UG=Hs.279694 hypothetical protein PRO1483/FL=gb:AF116635.1 gb:NM_018582.1	−4.72
213705_at	Methionine adenosyltransferase II, α	− −4.64
201438_at	Collagen, type VI, α3	− −4.59
217665_at	Consensus includes gb:AA420614/FEA=EST/DB_XREF=gi:2094586/DB_XREF= est: nc62g02.r1/CLONE=IMAGE:745874/UG=Hs.188826 ESTs, Moderately similar to G02654 ribosomal protein L39 *H. sapiens*	−4.17
209459_s_at	4-aminobutyrate aminotransferase	−3.99
220992_s_at	Chromosome 1 open reading frame 25///chromosome 1 open reading frame 25	−3.81
222294_s_at	Eukaryotic translation initiation factor 2C, 2	−3.78
221995_s_at	Consensus includes gb:BF195165/FEA=EST/DB_XREF=gi:11081754/DB_XREF= est: 7n16b01.×1/CLONE=IMAGE:3564624/UG=Hs.182695 hypothetical protein MGC3243	−3.71
215095_at	Esterase D/formylglutathione hydrolase	−3.68
212675_s_at	KIAA0582	−3.66
210187_at	FK506 binding protein 1A, 12 kDa	−3.65
204634_at	NIMA (never in mitosis gene a)-related kinase 4	−3.59
203791_at	Dmx-like 1	−3.53
205583_s_at	Chromosome X open reading frame 45	−3.53
218352_at	Regulator of chromosome condensation (RCC1) and BTB (POZ) domain containing protein 1	−3.52
209788_s_at	Type 1 tumor necrosis factor receptor shedding aminopeptidase regulator	−3.48
212959_s_at	MGC4170 protein	−3.47
205802_at	Transient receptor potential cation channel, subfamily C, member 1	−3.43
202732_at	Protein kinase (cAMP-dependent, catalytic) inhibitor γ	− −3.40
202149_at	Neural precursor cell expressed, developmentally downregulated 9	−3.39
213225_at	Protein phosphatase 1B (formerly 2C), magnesium-dependent, β isoform	−3.39
213624_at	Sphingomyelin phosphodiesterase, acid-like 3A	−3.39
207855_s_at	Mid-1-related chloride channel 1	−3.37
204415_at	Interferon, α-inducible protein (clone IFI-6-16)	−3.29
210017_at	Mucosa associated lymphoid tissue lymphoma translocation gene 1	−3.12
205420_at	Peroxisomal biogenesis factor 7	−3.05
219317_at	Polymerase (DNA directed) iota	−3.01
204176_at	Kelch-like ECT2 interacting protein	−3.00
203741_s_at	Adenylate cyclase 7	−2.95
205034_at	Cyclin E2	−2.94
204078_at	Synaptonemal complex protein SC65	−2.90
203881_s_at	Dystrophin (muscular dystrophy, Duchenne and Becker types)	−2.88
209717_at	Ecotropic viral integration site 5	−2.87
213473_at	BRCA1 associated protein	−2.86
215949_x_at	Immunoglobulin heavy constant *μ*	−2.83
205668_at	Lymphocyte antigen 75	−2.83
219688_at	Bardet-Biedl syndrome 7	−2.82
207845_s_at	Anaphase promoting complex subunit 10	−2.80
208920_at	Sorcin	−2.79
218002_s_at	Chemokine (C-X-C motif) ligand 14	−2.53
208727_s_at	Cell division cycle 42 (GTP binding protein, 25 kDa)	−2.25

**Table III:** Genes up- and downregulated by Ro25-7386 in MCF-7 cells.

A, Genes upregulated by Ro25-7386		
Probe set	Gene	Fold change
209909_s_at	Transforming growth factor, β2	4.94
211430_s_at	Immunoglobulin heavy constant γ 1 (G1m marker)	3.82
213010_at	Protein kinase C, δ binding protein	3.76
63825_at	Abhydrolase domain containing 2	3.40
208993_s_at	Peptidyl-prolyl isomerase G (cyclophilin G)	3.39
204681_s_at	Rap guanine nucleotide exchange factor (GEF) 5	3.26
213536_s_at	gb:AA910614/DB_XREF=gi:3049904/DB_XREF=ok61b04.s1/CLONE=IMAGE: 1518415/FEA=EST/CNT=42/TID=Hs.84285.2/TIER=Stack/STK=12/UG=Hs. 84285/LL=7329/UG_GENE=UBE2I/UG_TITLE=ubiquitin-conjugating enzyme E2I (homologous to yeast UBC9)	3.20
213087_s_at	Eukaryotic translation elongation factor 1 δ (guanine nucleotide exchange protein)	3.09
217489_s_at	Interleukin 6 receptor	3.07
205443_at	Small nuclear RNA activating complex, polypeptide 1, 43 kDa	3.04
213747_at	Consensus includes gb:AA047234/FEA=EST/DB_XREF=gi:1525134/DB_XREF=est:zf50b09.s1/CLONE=IMAGE:380345/UG=Hs.223014 antizyme inhibitor	2.99
221815_at	Abhydrolase domain containing 2	2.95
212451_at	KIAA0256 gene product	2.93
205363_at	Butyrobetaine (γ), 2-oxoglutarate dioxygenase (γ-butyrobetaine hydroxylase) 1	2.92
212952_at	Consensus includes gb:AA910371/FEA=EST/DB_XREF=gi:3049661/DB_XREF=est: ok83h10.s1/CLONE=IMAGE:1520611/UG=Hs.16488 calreticulin	2.90
210136_at	Myelin basic protein	2.88
214255_at	ATPase, class V, type 10A	2.87
213789_at	Consensus includes gb:N58493/FEA=EST/DB_XREF=gi:1202383/DB_XREF=est: yv72d01.s1/CLONE=IMAGE:248257/UG=Hs.75105 emopamil-binding protein (sterol isomerase)	2.86
217464_at	Consensus includes gb:L48784/DEF=050 Homo sapiens cDNA/FEA=mRNA/DB_XREF=gi:1066715/UG=Hs.182426 ribosomal protein S2	2.83
210841_s_at	Neuropilin 2	2.82
204378_at	Breast carcinoma amplified sequence 1	2.80
208859_s_at	α thalassemia/mental retardation syndrome X-linked (RAD54 homolog, *S. cerevisiae*)	2.76
221018_s_at	Tudor domain containing 1///tudor domain containing 1	2.76
218876_at	Brain specific protein///brain specific protein	2.73
215081_at	KIAA1024 protein	2.71
201510_at	E74-like factor 3 (ets domain transcription factor, epithelial-specific)	2.69
210089_s_at	Laminin, α4	2.68
218859_s_at	Chromosome 20 open reading frame 6	2.65
211626_x_at	v-ets erythroblastosis virus E26 oncogene like (avian)///v-ets erythroblastosis virus E26 oncogene like (avian)	2.64
214316_x_at	gb:AI378706/DB_XREF=gi:4188559/DB_XREF=tb91f09.×1/CLONE=IMAGE:2061737/FEA=EST/CNT=13/TID=Hs.16488.3/TIER=Stack/STK=13/UG=Hs.16488/LL=811/UG_GENE=CALR/UG_TITLE=calreticulin	2.64
220657_at	Kelch-like 11 (*Drosophila*)	2.61
206490_at	Discs, large (*Drosophila*) homolog-associated protein 1	2.60
208383_s_at	Phosphoenolpyruvate carboxykinase 1 (soluble)	2.59
214884_at	gb:AL033403/DB_XREF=gi:3859054/FEA=mRNA/CNT=15/TID=Hs.89543.1/TIER=ConsEnd/STK=0/UG=Hs.89543/LL=4168/UG_GENE=MCF2/UG_TITLE=MCF.2 cell line derived transforming sequence/DEF=Human DNA sequence from clone 88D7 on chromosome Xq25-26.3 Contains F9 (coagulation factor IX (plasma thromboplastic component, Christmas disease, haemophilia B)), dbl oncogene. EST, STS, GSS	2.59
201506_at	Transforming growth factor, β-induced, 68 kDa	2.18
213979_s_at	Consensus includes gb:BF984434/FEA=EST/DB_XREF=gi:12387246/DB_XREF=est: 602307971F1/CLONE=IMAGE:4399313/UG=Hs.239737 C-terminal binding protein 1	2.50
211253_x_at	Peptide YY	2.38
206879_s_at	Neuregulin 2	2.33
208835_s_at	Cisplatin resistance-associated overexpressed protein	2.33
201506_at	Transforming growth factor, β-induced, 68 kDa	2.18

B, Genes downregulated by Ro25-7386		
Probe set	Gene	Fold change

202901_x_at	Cathepsin S	−64.37
201367_s_at	Zinc finger protein 36, C3H type-like 2	−5.67
213606_s_at	Rho GDP dissociation inhibitor (GDI) α	−−5.01
211136_s_at	Cleft lip and palate associated transmembrane protein 1	−4.59
204989_s_at	Integrin, β4	−4.51
213042_s_at	ATPase, Ca++ transporting, ubiquitous	−4.42
216971_s_at	Plectin 1, intermediate filament binding protein 500 kDa	−4.37
201167_x_at	Rho GDP dissociation inhibitor (GDI) α	−4.14
219529_at	Chloride intracellular channel 3	−3.97
218813_s_at	SH3-domain GRB2-like endophilin B2	−3.93
211905_s_at	Integrin, β4	−3.87
211672_s_at	Actin related protein 2/3 complex, subunit 4, 20 kDa///actin related protein 2/3 complex, subunit 4, 20kDa	−3.70
207521_s_at	ATPase, Ca++ transporting, ubiquitous	−3.44
213986_s_at	Chromosome 19 open reading frame 6	−3.43
207824_s_at	MYC-associated zinc finger protein (purine-binding transcription factor)	−3.42
203085_s_at	Transforming growth factor, β1 (Camurati-Engelmann disease)	−3.34
203953_s_at	Claudin 3	−3.26
211019_s_at	Lanosterol synthase (2,3-oxidosqualene-lanosterol cyclase)	−3.22
209872_s_at	Plakophilin 3	−3.20
214326_x_at	Jun D proto-oncogene	−3.14
208677_s_at	Basigin (OK blood group)	−3.12
201245_s_at	OTU domain, ubiquitin aldehyde binding 1	−3.08
203751_x_at	Jun D proto-oncogene	−3.08
203370_s_at	PDZ and LIM domain 7 (enigma)	−3.05
203028_s_at	Cytochrome b-245, α polypeptide	−3.02
210954_s_at	KIAA0669 gene product	−2.99
211823_s_at	Paxillin	−2.97
200968_s_at	Peptidylprolyl isomerase B (cyclophilin B)	−2.93
205463_s_at	Platelet-derived growth factor α polypeptide	−2.87
210317_s_at	Tyrosine 3-monooxygenase/tryptophan 5-monooxygenase activation protein, ε polypeptide	−2.87
211300_s_at	Tumor protein p53 (Li-Fraumeni syndrome)	−2.84
214251_s_at	Nuclear mitotic apparatus protein 1	−2.81
207722_s_at	BTB (POZ) domain containing 2	−2.80
216969_s_at	Kinesin family member 22	−2.79
203809_s_at	v-akt murine thymoma viral oncogene homolog 2	−2.76
218848_at	Hypothetical protein MGC2655	−2.73
212090_at	Glutamate receptor, ionotropic, N-methyl D-asparate-associated protein 1 (glutamate binding)	−2.69
201373_at	Plectin 1, intermediate filament binding protein 500 kDa	−2.68
218302_at	Presenilin enhancer 2	−2.68
213887_s_at	Polymerase (RNA) II (DNA directed) polypeptide E, 25 kDa	−2.67
201369_s_at	Zinc finger protein 36, C3H type-like 2	
203392_s_at	C-terminal binding protein 1	−2.50
200796_s_at	Myeloid cell leukemia sequence 1 (BCL2-related)	−2.43
206665_s_at	BCL2-like 1	−2.35

**Table IV:** Genes up- and downregulated by LGD1069 in T47D cells.

A, Genes upregulated by LGD1069		
Probe set	Gene	Fold change
215653_at	Consensus includes gb:AF339805.1/DEF=Homo sapiens clone IMAGE:248602, mRNA sequence./FEA=mRNA/DB_XREF=gi:13507343/UG=Hs.326719 Homo sapiens clone IMAGE:248602, mRNA sequence	4.74
206424_at	Cytochrome P450, family 26, subfamily A, polypeptide 1	2.98
202481_at	Dehydrogenase/reductase (SDR family) member 3	2.79
211689_s_at	Transmembrane protease, serine 2///transmembrane protease, serine 2	2.61
213629_x_at	Metallothionein 1F (functional)	2.32
215924_at	Consensus includes gb:AK022102.1/DEF=Homo sapiens cDNA FLJ12040 fis, clone HEMBB1001944./FEA=mRNA/DB_XREF=gi:10433423/UG=Hs.296687 Homo sapiens cDNA FLJ12040 fis, clone HEMBB1001944	2.32
208581_x_at	Metallothionein 1X	2.31
210827_s_at	E74-like factor 3 (ets domain transcription factor, epithelial-specific)	2.28
217165_x_at	Metallothionein 1F (functional)	2.23
204326_x_at	Metallothionein 1X	2.19
206461_x_at	Metallothionein 1H	2.15
204470_at	Chemokine (C-X-C motif) ligand 1 (melanoma growth stimulating activity, α)	2.14
204745_x_at	Metallothionein 1G	2.13
217028_at	Chemokine (C-X-C motif) receptor 4	2.04
212185_x_at	Consensus includes gb:NM_005953.1/DEF=Homo sapiens metallothionein 2A (MT2A), mRNA./FEA=CDS/GEN=MT2A/PROD=metallothionein 2A/DB_XREF=gi:5174763/UG=Hs.118786 metallothionein 2A/FL=gb:NM_005953.1	2.02
211456_x_at	gb:AF333388.1/DB_XREF=gi:13310411/FEA=FLmRNA/CNT=1/TID=Hs.326774.0/TIER=FL/STK=0/UG=Hs.326774/DEF=Homo sapiens metallothionein 1H-like protein mRNA, complete cds./PROD=metallothionein 1H-like protein/FL=gb:AF333388.1	2.01

B, Genes downregulated by LGD1069		
Probe set	Gene	Fold change

207437_at	Neuro-oncological ventral antigen 1	−3.04
210806_at	KIAA0998	−2.33
202989_at	Regulator of G-protein signaling 1	−2.13

**Table V:** Genes up- and downregulated by LG100268 in T47D cells.

A, Genes upregulated by LG100268		
Probe set	Gene	Fold change
215653_at	Consensus includes gb:AF339805.1/DEF=Homo sapiens clone IMAGE:248602, mRNA sequence./FEA=mRNA/DB_XREF=gi:13507343/UG=Hs.326719 Homo sapiens clone IMAGE:248602, mRNA sequence	4.74
215924_at	Consensus includes gb:AK022102.1/DEF=Homo sapiens cDNA FLJ12040 fis, clone HEMBB1001944./FEA=mRNA/DB_XREF=gi:10433423/UG=Hs.296687 Homo sapiens cDNA FLJ12040 fis, clone HEMBB1001944	2.87
204470_at	Chemokine (C-X-C motif) ligand 1 (melanoma growth stimulating activity, α)	2.26

B, Genes downregulated by LG100268		
Probe set	Gene	Fold change
215655_at	Glutamate receptor, ionotropic, kainate 2	−3.29
220327_at	Colon carcinoma-related protein	−2.94
213910_at	Insulin-like growth factor binding protein 7	−2.66
207145_at	Growth differentiation factor 8	−2.49
210806_at	KIAA0998	−2.30

**Table VI:** Genes up- and downregulated by Ro25-7386 in T47D cells.

A, Genes upregulated by Ro25-7386		
Probe set	Gene	Fold change
215653_at	Consensus includes gb:AF339805.1/DEF=Homo sapiens clone IMAGE:248602, mRNA sequence./FEA=mRNA/DB_XREF=gi:13507343/UG=Hs.326719 Homo sapiens clone IMAGE:248602, mRNA sequence	4.74
215924_at	Consensus includes gb:AK022102.1/DEF=Homo sapiens cDNA FLJ12040 fis, clone HEMBB1001944./FEA=mRNA/DB_XREF=gi:10433423/UG=Hs.296687 Homo sapiens cDNA FLJ12040 fis, clone HEMBB1001944	2.87
204470_at	Chemokine (C-X-C motif) ligand 1 (melanoma growth stimulating activity, α)	2.26

B, Genes downregulated by Ro25-7386		
Probe set	Gene	Fold change

215655_at	Glutamate receptor, ionotropic, kainate 2	−3.29
220327_at	Colon carcinoma-related protein	−2.94
213910_at	Insulin-like growth factor binding protein 7	−2.66
207145_a	Growth differentiation factor 8	−2.49
210806_at	KIAA0998	−2.30

**Table VII:** Genes up- and downregulated by LGD1069 in MDA-MB-231.

A, Genes upregulated by LGD1069		
Probe set	Gene	Fold change
219948_x_at	Hypothetical protein FLJ21934	232.43
209672_s_at	Hypothetical protein FLJ20323	69.61
207750_at	gb:NM_018510.1/DEF=Homo sapiens hypothetical protein PRO1866 (PRO1866), mRNA. /FEA=mRNA/GEN=PRO1866/PROD=hypothetical protein PRO1866/DB_XREF=gi: 8924091/UG=Hs.283031 hypothetical protein PRO1866/FL=gb:AF119858.1 gb:NM_018510.1	30.50
203603_s_at	Zinc finger homeobox 1b	10.18
217698_at	Consensus includes gb:AV651668/FEA=EST/DB_XREF=gi:9872682/DB_XREF=est:AV651668/CLONE=GLCCSC04/UG=Hs.282480 ESTs	10.11
AFFX-r2-	E. coli/GEN=bioB/DB_XREF=gb:J04423.1/NOTE=SIF corresponding to nucleotides	9.76
Ec-bioB-	2393-2682 of gb:J04423.1/DEF=E.coli 7,8-diamino-pelargonic acid (bioA), biotin synthetase	
M_at	(bioB), 7-keto-8-amino-pelargonic acid synthetase (bioF), bioC protein, and dethiobiot	
205386_s_at	Mdm2, transformed 3T3 cell double minute 2, p53 binding protein (mouse)	9.65
216119_s_at	Chromosome 20 open reading frame 28	9.42
AFFX-	E. coli/GEN=bioB/DB_XREF=gb:J04423.1/NOTE=SIF corresponding to nucleotides	9.32
BioB-M_at	2482-2739 of gb:J04423.1/DEF=E.coli 7,8-diamino-pelargonic acid (bioA), biotin synthetase (bioB), 7-keto-8-amino-pelargonic acid synthetase (bioF), bioC protein, and dethiobiot	
209613_s_at	Alcohol dehydrogenase IB (class I), β polypeptide	8.85
AFFX-r2-	E. coli/GEN=bioB/DB_XREF=gb:J04423.1/NOTE=SIF corresponding to nucleotides	8.78
Ec-bioB-3_at	2772-3004 of gb:J04423.1/DEF=E.coli 7,8-diamino-pelargonic acid (bioA), biotin synthetase (bioB), 7-keto-8-amino-pelargonic acid synthetase (bioF), bioC protein, and dethiobiot	
217194_at	Consensus includes gb:AB007970.1/DEF=Homo sapiens mRNA, chromosome 1 specific transcript KIAA0501./FEA=mRNA/DB_XREF=gi:3413945/UG=Hs.223020 Homo sapiens mRNA, chromosome 1 specific transcript KIAA0501	7.08
205524_s_at	Hyaluronan and proteoglycan link protein 1	7.06
215514_at	Consensus includes gb:AL080072.1/DEF=Homo sapiens mRNA; cDNA DKFZp564M0616 (from clone DKFZp564M0616)./FEA=mRNA/DB_XREF=gi:5262482/UG=Hs.21195 Homo sapiens mRNA; cDNA DKFZp564M0616 (from clone DKFZp564M0616)	6.85
214774_x_at	Trinucleotide repeat containing 9	6.70
215526_at	Consensus includes gb:AL050145.1/DEF=Homo sapiens mRNA; cDNA DKFZp586C2020 (from clone DKFZp586C2020)./FEA=mRNA/DB_XREF=gi:4884356/UG=Hs.225986 Homo sapiens mRNA; cDNA DKFZp586C2020 (from clone DKFZp586C2020)	6.22
211091_s_at	Neurofibromin 2 (bilateral acoustic neuroma)	6.21
221959_at	Hypothetical protein MGC39325	6.11
206863_x_at	gb:U76376.1/DB_XREF=gi:1923234/GEN=HRK/FEA=FLmRNA/CNT=9/TID=Hs.87247.0/TIER=ConsEnd/STK=0/UG=Hs.87247/LL=8739/DEF=Homo sapiens activator of apoptosis Hrk (HRK) mRNA, complete cds./PROD=activator of apoptosis Hrk/FL=gb:NM_003806.1 gb:U76376.1	6.09
206202_at	Mesenchyme homeo box 2 (growth arrest-specific homeo box)	5.75
205288_at	CDC14 cell division cycle 14 homolog A (*S. cerevisiae*)	5.62
220931_at	Hypothetical protein MGC5590	5.40
216795_at	CDNA: FLJ23194 fis, clone REC00490	5.29
206410_at	Nuclear receptor subfamily 0, group B, member 2	5.23
207647_at	Chromodomain protein, Y-linked, 1///chromodomain protein, Y-linked, 1B	5.19
215112_x_at	MCF.2 cell line derived transforming sequence-like 2	5.11
216775_at	Ubiquitin specific protease 53	4.90
220109_at	Transferrin	4.88
217132_at	Clone 24587 mRNA sequence	4.86
216737_at	CDNA: FLJ20872 fis, clone ADKA02604	4.84
220036_s_at	Lipocalin-interacting membrane receptor	4.70
AFFX-r2-	E. coli/GEN=bioD/DB_XREF=gb:J04423.1/NOTE=SIF corresponding to nucleotides	4.66
Ec-bioD-3_at	5312-5559 of gb:J04423.1, not 100% identical/DEF=E.coli 7,8-diamino-pelargonic acid (bioA), biotin synthetase (bioB), 7-keto-8-amino-pelargonic acid synthetase (bioF), bioC pro	
220564_at	Chromosome 10 open reading frame 59	4.64
211611_s_at	Tenascin XB///tenascin XB///cAMP responsive element binding protein-like 1///cAMP responsive element binding protein-like 1	4.61
AFFX-	E. coli/GEN=bioD/DB_XREF=gb:J04423.1/NOTE=SIF corresponding to nucleotides	
BioDn-3_at	5286-5570 of gb:J04423.1, not 100% identical/DEF=E.coli 7,8-diamino-pelargonic acid (bioA), biotin synthetase (bioB), 7-keto-8-amino-pelargonic acid synthetase (bioF), bioC pro	4.49
207272_at	Zinc finger protein 80 (pT17)	4.49
210690_at	Killer cell lectin-like receptor subfamily C, member 4	4.47
216625_at	Consensus includes gb:AL050032.1/DEF=Homo sapiens mRNA; cDNA DKFZp566F1224 (from clone DKFZp566F1224)./FEA=mRNA/DB_XREF=gi:4884272/UG=Hs.306307 Homo sapiens mRNA; cDNA DKFZp566F1224 (from clone DKFZp566F1224)	4.37
207245_at	UDP glycosyltransferase 2 family, polypeptide B17	4.35
208014_x_at	Neuronal thread protein AD7c-NTP	4.32
214767_s_at	Heat shock protein, α-crystallin-related, B6	4.31
216697_at	Triple functional domain (PTPRF interacting)	4.28
222341_x_at	Consensus includes gb:AW973235/FEA=EST/DB_XREF=gi:8163081/DB_XREF=est: EST385333/UG=Hs.293697 ESTs	4.27
207262_at	Apolipoprotein F	4.25
222320_at	Consensus includes gb:AW970584/FEA=EST/DB_XREF=gi:8160429/DB_XREF=est: EST382665/UG=Hs.291033 ESTs	4.14
206201_s_at	Mesenchyme homeo box 2 (growth arrest-specific homeo box)	4.06
208019_at	Zinc finger protein 157 (HZF22)	4.01
204991_s_at	Neurofibromin 2 (bilateral acoustic neuroma)	3.97
207607_at	Achaete-scute complex-like 2 (Drosophila)	3.88
AFFX-r2-	E. coli/GEN=bioD/DB_XREF=gb:J04423.1/NOTE=SIF corresponding to nucleotides	3.83
Ec-bioD-5_at	5024-5244 of gb:J04423.1/DEF=E.coli 7,8-diamino-pelargonic acid (bioA), biotin synthetase (bioB), 7-keto-8-amino-pelargonic acid synthetase (bioF), bioC protein, and dethiobiot	
211315_s_at	Calcium channel, voltage-dependent, α 1G subunit	3.78
205953_at	Leucine-rich repeats and immunoglobulin-like domains 2	3.75
207781_s_at	Zinc finger protein 6 (CMPX1)	3.74
216068_at	Sodium- and chloride-activated ATP-sensitive potassium channel	3.69
214899_at	Hypothetical BC331191_1	3.59
208212_s_at	Anaplastic lymphoma kinase (Ki-1)	3.58

B, Genes downregulated by LGD1069		
Probe set	Gene	Fold change

215117_at	Recombination activating gene 2	−60.45
217535_at	Consensus includes gb:AV720514/FEA=EST/DB_XREF=gi:10817666/DB_XREF=est: - AV720514/CLONE=GLCGSB09/UG=Hs.282721 ESTs, Weakly similar to ALU7_HUMAN ALU SUBFAMILY SQ SEQUENCE CONTAMINATION WARNING ENTRY H.sapiens	16.22
201691_s_at	Tumor protein D52	−16.09
207674_at	Fc fragment of IgA, receptor for	−6.54
215172_at	DKFZP566K0524 protein	−5.85
218541_s_at	Chromosome 8 open reading frame 4	−5.79
215350_at	Spectrin repeat containing, nuclear envelope 1	−5.69
AFFX-HUMRGE/M10098_5_at	H. sapiens/GEN=18S rRNA/DB_XREF=gb:M10098.1/NOTE=SIF corresponding to nucleotides 115-595 of gb:M10098.1/DEF=Human 18S rRNA gene, complete.	−5.59
213652_at	Proprotein convertase subtilisin/kexin type 5	−5.57
216050_at	Transcribed locus, moderately similar to NP_803425.1 DNA segment, Chr 19, brigham & women’s genetics 1357 expressed [Mus musculus]	−5.43
222342_at	Consensus includes gb:AW979196/FEA=EST/DB_XREF=gi:8170484/DB_XREF=est: - EST391306/UG=Hs.292713 ESTs, Moderately similar to ALU1_HUMAN ALU SUBFAMILY J SEQUENCE CONTAMINATION WARNING ENTRY H.sapiens	5.41
205638_at	Brain-specific angiogenesis inhibitor 3	−5.04
217464_at	Consensus includes gb:L48784/DEF=050 Homo sapiens cDNA/FEA=mRNA/DB_XREF=gi:1066715/UG=Hs.182426 ribosomal protein S2	−4.97
205848_at	Growth arrest-specific 2	−4.86
206588_at	Deleted in azoospermia-like	−4.75
213826_s_at	Consensus includes gb:AA292281/FEA=EST/DB_XREF=gi:1940261/DB_XREF= est:zt51b03.s1/CLONE=IMAGE:725837/UG=Hs.181307 H3 histone, family 3A	−4.74
220432_s_at	Cytochrome P450, family 39, subfamily A, polypeptide 1	−4.48
209227_at	Tumor suppressor candidate 3	−4.41
211712_s_at	Annexin A9///annexin A9	−4.31
AFFX-HUMRGE/M10098_M_at	H. sapiens/GEN=18S rRNA/DB_XREF=gb:M10098.1/NOTE=SIF corresponding to nucleotides 688-1219 of gb:M10098.1/DEF=Human 18S rRNA gene, complete.	−4.28
AFFX-HUMRGE/M10098_3_at	Signal recognition particle 68 kDa	−4.20
202648_at	gb:BC000023.1/DB_XREF=gi:12652562/FEA=FLmRNA/CNT=966/TID=Hs.298262. - 0/TIER=ConsEnd/STK=0/UG=Hs.298262/LL=6223/UG_GENE=RPS19/DEF= Homo sapiens, ribosomal protein S19, clone MGC:1630, mRNA, complete cds./PROD= ribosomal protein S19/FL=gb:M81757.1 g	4.15
207815_at	Platelet factor 4 variant 1	−4.15
205363_at	Butyrobetaine (γ), 2-oxoglutarate dioxygenase (γ-butyrobetaine hydroxylase) 1	−4.14
213856_at	CD47 antigen (Rh-related antigen, integrin-associated signal transducer)	−4.11
216087_at	MRNA full length insert cDNA clone EUROIMAGE 117929	−4.11
211264_at	Glutamate decarboxylase 2 (pancreatic islets and brain, 65 kDa)	−4.03
220771_at	Melanoma antigen	−3.83
220474_at	Solute carrier family 25 (mitochondrial oxodicarboxylate carrier), member 21	−3.81
220281_at	Solute carrier family 12 (sodium/potassium/chloride transporters), member 1	−3.80
217524_x_at	Consensus includes gb:AA018923/FEA=EST/DB_XREF=gi:1482314/DB_XREF= - est:ze58d03.s1/CLONE=IMAGE:363173/UG=Hs.261204 ESTs	3.72
211776_s_at	Erythrocyte membrane protein band 4.1-like 3///erythrocyte membrane protein band 4.1-like 3	−3.69
212681_at	Erythrocyte membrane protein band 4.1-like 3	−3.69
217333_at	Consensus includes gb:AL031903/DEF=Human DNA sequence from clone 1032F13 on chromosome Xq25-26.3. Contains a pseudogene similar to Keratin 18 (KRT18, Cytokeratin 18) and ESTs/FEA=CDS/DB_XREF=gi:3766260/UG=Hs.247763 Human DNA sequence from clone 1032F1	−3.69
210721_s_at	p21(CDKN1A)-activated kinase 7	−3.63
210327_s_at	Alanine-glyoxylate aminotransferase (oxalosis I; hyperoxaluria I; glycolicaciduria; serine-pyruvate aminotransferase)	−3.57
206265_s_at	Glycosylphosphatidylinositol specific phospholipase D1	−3.54
205847_at	Protease, serine, 22	−3.52
202901_x_at	Cathepsin S	−3.42
204681_s_at	Rap guanine nucleotide exchange factor (GEF) 5	−3.35
222227_at	Zinc finger protein 236	−3.35
207465_at	PRO0628 protein	−3.34

**Table VIII:** Genes upregulated and downregulated by LG100268 in MDA-MB-231 cells.

A, Genes upregulated by LG100268 in MDA-MB-231		
Probe set	Gene	Fold change
219948_x_at	Hypothetical protein FLJ21934	88.95
207750_at	gb:NM_018510.1/DEF=Homo sapiens hypothetical protein PRO1866 (PRO1866), mRNA./FEA=mRNA/GEN=PRO1866/PROD=hypothetical protein PRO1866/DB_XREF=gi:8924091/UG =Hs.283031 hypothetical protein PRO1866/FL=gb:AF119858.1 gb:NM_018510.1	26.42
209672_s_at	Hypothetical protein FLJ20323	14.63
215514_at	Consensus includes gb:AL080072.1/DEF=Homo sapiens mRNA; cDNA DKFZp564M0616 (from clone DKFZp564M0616)./FEA=mRNA/DB_XREF=gi:5262482/UG=Hs.21195 Homo sapiens mRNA; cDNA DKFZp564M0616 (from clone DKFZp564M0616)	9.11
215309_at	Transcribed locus, weakly similar to XP_092995.4 zinc finger protein 21 (KOX 14) [*Homo sapiens*]	8.12
214774_x_at	Trinucleotide repeat containing 9	7.58
203603_s_at	Zinc finger homeobox 1b	5.77
205386_s_at	Mdm2, transformed 3T3 cell double minute 2, p53 binding protein (mouse)	5.20
205419_at	Epstein-Barr virus induced gene 2 (lymphocyte-specific G protein-coupled receptor)	4.18
216978_x_at	Consensus includes gb:U50277.1/DEF=Human breast cancer suppressor element Ishmael Upper CP1 mRNA, partial cds./FEA=mRNA/PROD=suppressor element Ishmael Upper CP1/DB_XREF=gi:1224126/UG=Hs.121485 Human breast cancer suppressor element Ishmael Upper CP	3.93
220931_at	Hypothetical protein MGC5590	3.81
219995_s_at	Hypothetical protein FLJ13841	3.77
208076_at	Histone 1, H4d	3.6
214255_at	ATPase, Class V, type 10A	3.55
207987_s_at	Gonadotropin-releasing hormone 1 (luteinizing-releasing hormone)	3.52
205651_x_at	Rap guanine nucleotide exchange factor (GEF) 4	3.46
220401_at	Hypothetical protein FLJ21369	3.39
207241_at	Chromosome 4 open reading frame 6	3.35
215623_x_at	SMC4 structural maintenance of chromosomes 4-like 1 (yeast)	3.17
216119_s_at	Chromosome 20 open reading frame 28	3.13
217194_at	Consensus includes gb:AB007970.1/DEF=Homo sapiens mRNA, chromosome 1 specific transcript KIAA0501./FEA=mRNA/DB_XREF=gi:3413945/UG=Hs.223020 Homo sapiens mRNA, chromosome 1 specific transcript KIAA0501	3.10
206381_at	Sodium channel, voltage-gated, type II, α 2	3.09
212182_at	Nudix (nucleoside diphosphate linked moiety X)-type motif 4	2.98
215112_x_at	MCF.2 cell line derived transforming sequence-like 2	2.94
213747_at	Consensus includes gb:AA047234/FEA=EST/DB_XREF=gi:1525134/DB_XREF= est:zf50b09.s1/CLONE=IMAGE:380345/UG=Hs.223014 antizyme inhibitor	2.84
221683_s_at	Centrosome protein cep290	2.80
211611_s_at	Tenascin XB///tenascin XB///cAMP responsive element binding protein-like 1///cAMP responsive element binding protein-like 1	2.74
205421_at	Solute carrier family 22 (extraneuronal monoamine transporter), member 3	2.66
213764_s_at	Microfibrillar associated protein 5	2.62
217505_at	Hypothetical protein MGC22679	2.61
222320_at	Consensus includes gb:AW970584/FEA=EST/DB_XREF=gi:8160429/DB_XREF=est: EST382665/UG=Hs.291033 ESTs	2.61
216466_at	Neuron navigator 3	2.59
AFFX-r2-	E. coli/GEN=bioB/DB_XREF=gb:J04423.1/NOTE=SIF corresponding to nucleotides	2.55
Ec-bio	2393-2682 of gb:J04423.1/DEF=E.coli 7,8-diamino-pelargonic acid (bioA), biotin synthetase	
B-M_at	(bioB), 7-keto-8-amino-pelargonic acid synthetase (bioF), bioC protein, and dethiobiot	
216775_at	Ubiquitin specific protease 53	2.54
206201_s_at	Mesenchyme homeo box 2 (growth arrest-specific homeo box)	2.53
AFFX-	E. coli/GEN=bioD/DB_XREF=gb:J04423.1/NOTE=SIF corresponding to nucleotides	2.48
BioDn-5_at	4980-5256 of gb:J04423.1, not 100% identical/DEF=E.coli 7,8-diamino-pelargonic acid (bioA), biotin synthetase (bioB), 7-keto-8-amino-pelargonic acid synthetase (bioF), bioC pro	
216894_x_at	Cyclin-dependent kinase inhibitor 1C (p57, Kip2)	2.46
208019_at	Zinc finger protein 157 (HZF22)	2.45
215803_at	Hypothetical protein FLJ10178	2.44
222320_at	CDNA: FLJ23194 fis, clone REC00490	2.44

B, Genes downregulated by LG100268		
Probe set	Gene	Fold change

217237_at	Zinc finger protein 423	−78.6
215014_at	Consensus includes gb:AL512727.1/DEF=Homo sapiens mRNA; cDNA DKFZp547P042 (from clone DKFZp547P042)./FEA=mRNA/DB_XREF=gi:12224870/UG=Hs.232127 Homo sapiens mRNA; cDNA DKFZp547P042 (from clone DKFZp547P042)	−17.74
213753_x_at	Eukaryotic translation initiation factor 5A	−7.65
212382_at	Transcription factor 4	−5.74
AFFX-HUMRGE/M10098_5_at	H. sapiens/GEN=18S rRNA/DB_XREF=gb:M10098.1/NOTE=SIF corresponding to nucleotides 115-595 of gb:M10098.1/DEF=Human 18S rRNA gene, complete	−5.58
211712_s_at	Annexin A9///annexin A9	−5.49
209227_at	Tumor suppressor candidate 3	−5.11
216917_s_at	Synaptonemal complex protein 1	−4.82
AFFX-HUMRGE/M10098_M_at	H. sapiens/GEN=18S rRNA/DB_XREF=gb:M10098.1/NOTE=SIF corresponding to nucleotides 688-1219 of gb:M10098.1/DEF=Human 18S rRNA gene, complete	−4.31
210697_at	Zinc finger protein 257	−4.11
215013_s_at	Ubiquitin specific protease 34	−3.97
209657_s_at	Heat shock transcription factor 2	−3.96
221009_s_at	Angiopoietin-like 4	−3.90
205612_at	Multimerin 1	−3.79
207613_s_at	Calcium/calmodulin-dependent protein kinase (CaM kinase) II α	−3.55
37232_at	KIAA0586	−3.38
AFFX-HUMRGE/M10098_3_at	Signal recognition particle 68 kDa	−3.37
204422_s_at	Fibroblast growth factor 2 (basic)	−3.33
220638_s_at	Cas-Br-M (murine) ecotropic retroviral transforming sequence c	−3.32
208098_at	Olfactory receptor, family 12, subfamily D, member 3///olfactory receptor, family 12, subfamily D, member 3///olfactory receptor, family 5, subfamily V, member 1///olfactory receptor, family 5, subfamily V, member 1	−3.29
213826_s_at	Consensus includes gb:AA292281/FEA=EST/DB_XREF=gi:1940261/DB_XREF - =est:zt51b03.s1/CLONE=IMAGE:725837/UG=Hs.181307 H3 histone, family 3A	3.25
208453_s_at	X-prolyl aminopeptidase (aminopeptidase P) 1, soluble	−3.20
207485_x_at	Butyrophilin, subfamily 3, member A1	−3.18
211032_at	COBL-like 1///COBL-like 1	−3.11
220619_at	Chromodomain helicase DNA binding protein 7	−3.04
209318_x_at	Pleiomorphic adenoma gene-like 1	−3.00
201547_at	Jumonji, AT rich interactive domain 1B (RBP2-like)	−2.99
206996_x_at	Calcium channel, voltage-dependent, β1 subunit	−2.98
220114_s_at	Stabilin 2	−2.95
216709_at	Hypothetical gene supported by BC013370; BC034583	−2.93
203555_at	Protein tyrosine phosphatase, non-receptor type 18 (brain-derived)	−2.92
13267_at	KIAA1117	−2.91
201122_x_at	Eukaryotic translation initiation factor 5A	−2.89
213495_s_at	gb:AW166067/DB_XREF=gi:6397592/DB_XREF=xf44g10.×1/CLONE=IMAGE: - 2620962/FEA=EST/CNT=75/TID=Hs.98614.2/TIER=Stack/STK=51/UG=Hs.98614/LL=6238/UG_GENE=RRBP1/UG_TITLE=ribosome binding protein 1 (dog 180kD homolog)	2.89
220301_at	Chromosome 18 open reading frame 14	−2.88
214837_at	Albumin	−2.85
209700_x_at	Phosphodiesterase 4D interacting protein (myomegalin)	−2.84
216805_at	Transcribed locus, moderately similar to XP_375099.1 hypothetical protein LOC283585 [*Homo sapiens*]	−2.84
221671_x_at	Immunoglobulin κ constant	−2.79
214001_x_at	gb:AW302047/DB_XREF=gi:6711724/DB_XREF=xr52f08.×1/CLONE=IMAGE:2763783/ - FEA=EST/CNT=24/TID=Hs.76230.2/TIER=Stack/STK=20/UG=Hs.76230/LL=6204/UG_GENE=RPS10/UG_TITLE=ribosomal protein S10	2.72
210047_at	Solute carrier family 11 (proton-coupled divalent metal ion transporters), member 2	−2.69
208367_x_at	Cytochrome P450, family 3, subfamily A, polypeptide 4	−2.66
219252_s_at	Family with sequence similarity 51, member A1	−2.65
205827_at	Cholecystokinin	−2.63
